# Supplementary material for: Pathophysiological Significance of Neutrophilic Transfer RNA-Derived Small RNAs in Asymptomatic Moyamoya Disease
Source: Cells. 2021 May 1;10(5):1086. doi: 10.3390/cells10051086 (PMC8147334; doi:10.3390/cells10051086)

2D Structure	Local AU	Position

```
2195                      7mer-m8  2223
5'-cttTGTGAAAGCTGCTATTTTACGGAAAg-3' utr
      :|||| |:|::|||: :||||||||
3'-uuuGCACUAUUGGUGAUGUGAUGCCUUUg-5' tRF
               16151413  765432
              3' Pairing   Seed
```

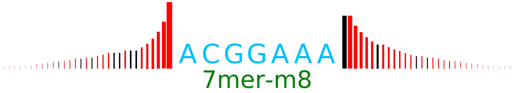

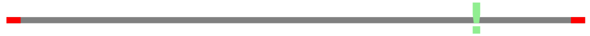

Supplement: Supplementary file 1 [file cells-10-01086-s001.zip › cells-1149161 3rd supplementary materials/Data Supplements/Data Supplement V.zip!/Data supplement 6-mRNAs targets of tsRNAs/tsRNA-Val-AAC-017/Diagrams/tRF-Val-AAC-017_vs_ENST00000381337(THUMPD1).pdf]
